# Successful Outcome of Uterocutaneous Fistula: A Case Report

**DOI:** 10.31729/jnma.7034

**Published:** 2021-09-30

**Authors:** Shilpi Mahto, Roshan Ghimire, Sarjan Kunwar, Rachana Saha

**Affiliations:** 1 Department of Obstetrics and Gynecology, Kathmandu Medical College and Teaching Hospital, Sinamangal, Kathmandu, Nepal; 2 Department of Surgery, Kathmandu Medical College and Teaching Hospital, Sinamangal, Kathmandu, Nepal

**Keywords:** *case report*, *cesarean section*, *uterocutaneous fistula*

## Abstract

Uterocutaneous fistula is a rare complication that occurs after cesarean section and other pelvic operations. Here we report a case of a 27 years woman presented to our department with a mass and pus-like discharge coming from her previous Pfannenstiel incision for 1 month. The definitive treatment of such cases is hysterectomy but the case was managed by fistulectomy along with gonadotropin-releasing hormone agonist.

## INTRODUCTION

Uterocutaneous fistula is a rare condition where communication is between uterus and skin.^1^ Multiple abdominal surgeries, endometriosis, and wound dehiscence have been implicated in the development of the fistula of the uterus.^2^ All published cases till date, have been managed either surgically with a hysterectomy and/or excision of a fistulous tract or medically by Gonadotropin-releasing hormone agonist. We report a very rare case of post-cesarean uterocutaneous fistula that was successfully treated with excision of the fistulous tract from the skin and repair of the fistulous uterus.

## CASE REPORT

A 27 years old woman presented to our department with mass and pus-like discharge coming from her previous Pfannenstiel incision for 1 month. She underwent lower segment cesarean section (LSCS) at a different hospital for fetal distress 6 months back. She was treated conservatively but she didn't improve. Ultrasound (USG) and Magnetic resonance imaging (MRI) were done which reported an 11-12mm fistulous tract extending through the muscular plane of the uterus. She was scheduled for a laparotomy but a patient came to our center for a second opinion. After counseling, the patient was planned for laparotomy with excision of the fistula tract and/or hysterectomy if required. Surgery was performed with a team of gynecologists and general surgeons. Intraoperatively, Methylene Blue dye was injected from the fistula opening and the tract was followed. Excision of the fistula tract was done and an 8f foley catheter was kept in the uterine cavity for 2 weeks. The immediate post-operative conditions were satisfactory. On the 10^th^ post-op day, the patient developed a Surgical site infection (SSI) and managed with daily dressing, oral antibiotics, and resuturing done after 2 weeks. She commenced on gonadotropin-releasing hormone agonist (GnRH) analog i.e. injection Leuprolide acetate 3.75mg intramuscular monthly for 6 months. Follow-up USG done after 1 month, showed no fistula tract and she was kept on monthly visits till 6 months where she had no complaints.

**Figure 1:**
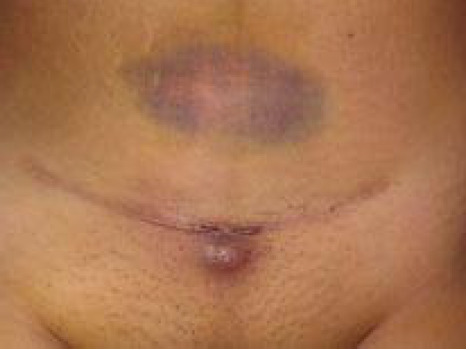
Fistula opening in the skin.

**Figure 2:**
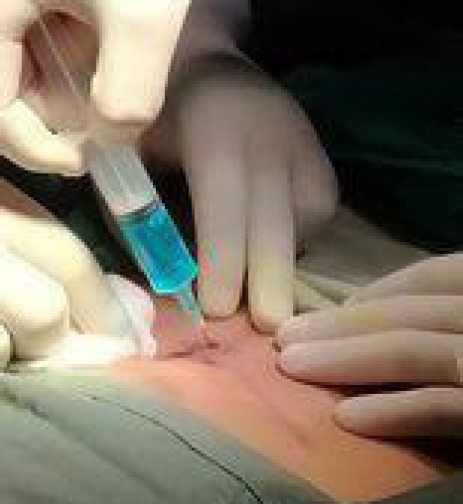
Injection of dye.

**Figure 3:**
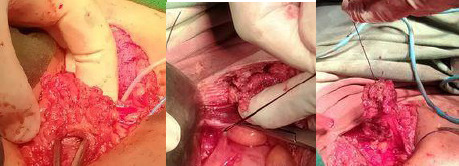
Tract followed, identified, and excised.

## DISCUSSION

Uterocutaneous fistula is an abnormal communication between the epithelial lining of the skin and uterus.^3^ It is a rare condition where the pathophysiology has not been clearly defined but documented following lower segment cesarean section, Infection, Inflammation, Postpartum sepsis, Invasive endometriosis, and uterine procedures.^4^ Thakur, et al. reported a 30-year old lady, who developed uterocutaneous fistula after undergoing emergency lower segment cesarean section for failed induction 2 months back and developed a massive primary postpartum hemorrhage at the time of cesarean section which was managed with B-Lynch suture and vessel ligation^5^ and Lim, et al.^6^ reported a 33-year-old lady who had an emergency cesarean section for retained second twin which was complicated by utero-cutaneous fistula due to red degeneration of intramural fibroids.

Jain, et al.^7^ reported uterocutaneous fistula following lower segment cesarean section.

Gupta, et al.^8^ reported uterocutaneous fistula which developed following septic abortion induced by laminaria tent insertion in the cervix. Because of the rarity of uterocutaneous fistula, there is no available evidence-based treatment modality. In the literature, the delay between last surgical event and occurrence of fistula ranges between 2 months to 6 years. The diagnosis of uterine fistula can be straightforward with pathognomonic clinical signs such as bloody discharge through abdominal scar during menstruation. Other investigations include - Fistulography with Injection of contrast material through skin opening showing connection to the uterus.^1^

Hysterosalpingography and MRI with contrast is another modality.^1^ A standard treatment has yet to be introduced. Recent studies suggest combining surgical and medical treatment to avoid the risk of hysterectomy.

In a case report of endometriotic uterocutaneous fistula following cesarean section, Dragoumis, et al. carried out a total abdominal hysterectomy in a 44 years old lady.^9^ However in another case report of uterocutaneous fistula following term abdominal pregnancy, promsonthi and Herbutya performed a subtotal hysterectomy.^10^ Seyhan, et al.^11^ reported a patient treated with GnRH agonist alone which induces atrophic changes in epithelium and assist in the closure of the fistula. Thubert, et al.^1^ used medical treatment and minimally invasive surgery for excision of the fistula tract. Although there is no evidence-based treatment modality currently available for utero-cutaneous fistula, various treatment options have been reported in the management of utero-cutaneous fistula where surgery has remained the treatment of choice. A combination of both medical and surgical treatment can be advantageous and averts the risks associated with major surgeries.^3^

This case report highlights the very rare possibility of uterocutaneous fistula occurring in a woman following cesarean section one month back. Appropriate surgical skills and postoperative care are necessary to prevent an outcome that may be agonizing for the patient. While there is no evidence-based modality of therapy, further research is necessary to explicate the mechanisms of this fistula following cesarean section. Imaging studies are instrumental in the evaluation of cases of uterocutaneous fistula.
